# Effects of 4-Week Training Intervention with Unknown Loads on Power Output Performance and Throwing Velocity in Junior Team Handball Players

**DOI:** 10.1371/journal.pone.0157648

**Published:** 2016-06-16

**Authors:** Rafael Sabido, Jose Luis Hernández-Davó, Javier Botella, Manuel Moya

**Affiliations:** Sports Research Centre, Miguel Hernández University, Elche, Spain; University of Rome Foro Italico, ITALY

## Abstract

**Purpose:**

To compare the effect of 4-week unknown vs known loads strength training intervention on power output performance and throwing velocity in junior team handball players.

**Methods:**

Twenty-eight junior team-handball players (17.2 ± 0.6 years, 1.79 ± 0.07 m, 75.6 ± 9.4 kg)were divided into two groups (unknown loads: UL; known loads: KL). Both groups performed two sessions weekly consisting of four sets of six repetitions of the bench press throw exercise, using the 30%, 50% and 70% of subjects’ individual 1 repetition maximum (1RM). In each set, two repetitions with each load were performed, but the order of the loads was randomised. In the KL group, researchers told the subjects the load to mobilise prior each repetition, while in the UL group, researchers did not provide any information. Maximal dynamic strength (1RM bench press), power output (with 30, 50 and 70% of 1RM) and throwing velocity (7 m standing throw and 9 m jumping throw) were assessed pre- and post-training intervention.

**Results:**

Both UL and KL group improved similarly their 1RM bench press as well as mean and peak power with all loads. There were significant improvements in power developed in all the early time intervals measured (150 ms) with the three loads (30, 50, 70% 1RM) in the UL group, while KL only improved with 30% 1RM (all the time intervals) and with 70% 1RM (at certain time intervals). Only the UL group improved throwing velocity in both standing (4.7%) and jumping (5.3%) throw (p > 0.05).

**Conclusions:**

The use of unknown loads has led to greater gains in power output in the early time intervals as well as to increases in throwing velocity compared with known loads. Therefore unknown loads are of significant practical use to increase both strength and in-field performance in a short period of training.

## Introduction

Power output production is an important component in many athletic activities. Therefore, the athletes’ ability to generate higher power output may determine sporting success [1]. The relationship between power and dynamic athletic performance has been well established in that muscular power is considered one of the main factors involved in sports that entail high force production over a short time [2,3]. In this regard, improvements in maximal power output have been accompanied by increased performance in several athletic pursuits such as jumping, sprinting and agility tests [4].

Among the sports that are related to the application of high levels of force in short periods of time, team handball is highly power-dependent since it involves high-intensity muscular actions such as sprinting, jumping and throwing [5]. Hermassi et al. [6] showed significant improvements in both standing and jumping throw velocities after 10 weeks of heavy load strength training, but the moderate load group only improved the velocity in the jumping throw. Moreover, Hermassi et al. [7] showed improvements in jumping height, sprinting performance and throwing velocities after 8 weeks of heavy-loads strength training in elite team handball players. Therefore, strength training seems to be a key point to improve specific team handball performance.

As a result, a wide number of studies have sought to improve muscle power performance through different strength training methodologies such as plyometrics [8], heavy loads [9], optimal loads [10] or the combination of heavy and light loads [11]. The theory behind the use of a combination of loads in a power training programme is to target all areas of the force-velocity relationship attempting to increase power adaptations throughout the entire curve. Thus, it is argued that training with a combination of loads may provide all-round improvements in the force-velocity relationship, which result in superior increases in maximal power output when compared with either light or heavy training loads alone [12].

In addition, the use of ballistic exercises (e.g. bench press throw) has shown greater power developments compared with traditional strength exercises [3,13]. The underlying mechanisms leading to power improvements when using ballistic exercises are not clearly defined. It is possible that these movements elicit adaptations in neural drive, the rate of neural activation, and inter-muscular coordination, which are commonly encountered in sports movements [3]. The results of these adaptations involve increases in the ability to generate more force in shorter periods of time [8,14]. Some of athletic skills (e.g. throwing, kicking, jumping) involve contraction times shorter than 250 ms, therefore, ballistic exercises are generally more sport-specific in a vast number of sports, allowing greater transference to performance. Supporting this is research showing significant improvements in maximal power output during sports-specific movements after a training programme with ballistic exercises [4,10,13,15]. Despite the wide number of methodologies found in the literature aiming at improving rapid force/power production, there is an obvious need among coaches and researchers to find new methods that could optimize improvements in such variables.

It has been recently shown that concentric-only bench press throws performed without knowledge about the load lifted entailed greater power output in the early phases from movement onset [16]. It is suggested that when preparing for a lift, the lifter makes assumptions regarding the weight of the load. If the load characteristics are not known or incorrectly judged, lifts are likely to be done more rapidly [17]. Thus, Marras et al. [18] found that the increased muscle response under unexpected condition was equivalent to doubling the load from the expected condition. In addition, the authors found that the unexpected condition elicits more rapid increases in trunk force development. Nevertheless, no studies have evaluated if these acute responses produced by using unexpected/unknown loads may result in greater neuromuscular adaptations after a training intervention consisting of unknown loads.

Based on previous studies showing improvements in power performance and team handball throwing velocities after strength training interventions, and the increased acute EMG response and greater power developed by performing explosive movements with lack of load knowledge, it was hypothesized that a strength training intervention based on unknown loads would entail larger improvements in team handball performance compared with traditional strength training.

## Materials and Methods

### Subjects

Twenty-eight elite junior team handball players volunteered to participate in this study. All were familiar with the bench press throw exercise as a part of their weekly strength training and previous testing. Descriptive data of each group are shown in Table 1. All subjects had at least 7 years experience in team handball, and at least 1 year experience in resistance training. Throughout the investigation, subjects were instructed to maintain their normal life habits. In addition, subjects were requested to maintain their regular diets and normal hydration state, not to take any nutritional supplementation or anti-inflammatory medications, and to refrain from caffeine intake in the 3 hours before each testing session. All training and testing sessions were scheduled the same days of the week and the same hours for each subject. Before participation, each subject, as well as their guardians were informed about the experimental risks and provided written informed consent (S1 File). The consent procedure and study (S2 File) were approved by the Ethics Committee of the University Miguel Hernández of Elche in accordance with the Declaration of Helsinki.

**Table 1:** Descriptive data of each group.

Group	Age (years)	Height (m)	Weight (kg)	1RM bench press (kg)
**UL**	17.4 ± 0.5	1.80 ± 0.07	76.3 ± 9.9	76.2 ± 11.9
**KL**	17.1 ± 0.6	1.78 ± 0.06	76.5 ± 9.8	74.6 ± 11.4
**C**	17.0 ± 0.6	1.76 ± 0.05	72.1 ± 8.1	72.8 ± 9.8

### Design

A randomised controlled study was designed to compare the effects of two different strength training interventions (known loads (KL); unknown loads (UL)) on strength parameters (1-RM and power performance in the bench press throw exercise) and specific throwing performance (7 m standing throw and 9 m jumping throw velocity). During the 4-week training intervention, both KL and UL group performed a specific bench press throw training added to their usual training two times per week (completing a total of 8 training sessions each group). In addition, a control group (C) who performed the usual training was also included. Pre-testing was carried out the week before starting the training intervention, while post-testing was conducted the week after finishing the 4 week training intervention. To avoid diurnal variation in training and testing measures, subjects were scheduled at approximately the same time for each testing and training sessions.

### Methodology

Tests were conducted at two different time points (the week before and following the 4-week training intervention). Subjects were required to attend 3 testing sessions on each time point, separated by 48h. On the first day, the 1RM test for the bench press exercise was carried out. On the second day, subjects performed the bench press throw power performance test. Both the first and second testing sessions were performed using a Smith Machine (Technogym, Gambettola, Italy). On the third day, subjects carried out the throwing velocity test. Testing selection was made based on the importance of both maximal strength and power output, as well as handball throwing velocity on handball performance [6,7].

### Maximal dynamic strength

The 1-RM bench press was assessed using a previously established protocol [19], which requires that subjects progressively increase resistance across attempts until the 1RM is achieved. Rest period between trials was at least 5 minutes. Subjects began by lying horizontally with the buttocks, lower back, upper back and head firmly planted on the bench, with elbows fully extended and gripping the bar. Subjects lowered the bar until the chest was slightly touched, approximately 3 cm superior to the xiphoid process. The elbows were extended equally with the head and hips remaining in contact with the bench, and the feet in contact with the floor throughout the lift. No bouncing or arching of the back was allowed.

### Bench press throw power performance

Three minutes after a warm-up consisting of two sets of 10 repetitions with the individuals 50% of 1RM, power performance was tested by performing one set of six repetitions of the bench press throw exercise using the individuals 30%, 50% and 70% 1RM. Rest period between sets was 5 minutes. An isoinertial dynamometer (T-Force Dynamic Measurement System, Ergotech, Murcia, Spain) was used for mechanical measurements. This system consists of a linear velocity transducer interfaced to a personal computer by means of a 14-bit resolution analog-to-digital data acquisition board, and custom software. Vertical instantaneous velocity was directly sampled by the device at a frequency of 1000 Hz. Instantaneous mechanical power output (P) was calculated as the product of vertical force and bar velocity (P = F · V). Peak power was taken as the maximum value of the power-time curve. The validity and reliability of this system have been previously established [20]. All data were saved to disk for subsequent analysis. The variables analysed were: peak power, mean power, and power in the early time intervals (30, 50, 100, and 150 ms) of the concentric phase.

### Throwing velocity

Handball throwing velocity was measured using a portable radar (Stalker sport 2, Applied Concepts Inc, USA) with an accuracy of 0.1 km·h^-1^. After 10 minutes of warm-up consisting of jogging, dynamic stretches and technical skills (passes and throws at submaximal velocities), each subject performed 3 maximal velocity throw attempts from both 7 m (standing throw) and 9 m (jumping throw). The standing throw has been described previously by Hermassi et al. [17]. In the jumping throw, players made a preparatory 3 step run before jumping vertically and releasing the ball while in the air, behind a line 9 m from the goal. The fastest throw of each throwing type was used for statistical analysis.

### Training

After the pre-testing sessions, subjects were randomly divided into two different training intervention (KL (n = 12) vs UL (n = 11)) and a control group (n = 5). Both experimental groups performed two sessions weekly consisting of four sets of six repetitions of the bench press throw exercise. The loads used were 30%, 50% and 70% of subjects’ individual 1RM. In each set, two repetitions with each load were performed, but the order of the loads was randomised (see Table 2). The rest interval between sets was three minutes.

**Table 2:** Example of a training session schedule.

	1^st^ rep	2^nd^ rep	3^rd^ rep	4^th^ rep	5^th^ rep	6^th^ rep
**Set 1**	30%	70%	50%	50%	70%	30%
**Set 2**	70%	50%	30%	70%	30%	50%
**Set 3**	50%	30%	70%	30%	50%	70%
**Set 4**	70%	30%	50%	50%	70%	30%

During the training sessions, the bench press throw movement was performed as follows: researchers let the barbell fall and subjects were instructed to decelerate it and then throw it as high as possible in an explosive ballistic movement. Then, when the barbell started to descend, researchers caught it and stopped in the Smith machine. Repetitions were executed in both groups every 10 seconds (as controlled by a metronome), during which the load was modified.

In the KL group, the main researcher told the subjects the load to mobilise before dropping the barbell, while in the UL group researchers dropped the barbell without providing any load information. To ensure subjects did not know the load in the UL, they performed the sets wearing special glasses. These glasses only allowed central sight, thus, subjects were able to see the centre of the barbell, but they did not see how the load was being manipulated. In addition, to guarantee that participants did not hear any sound when the load was being manipulated, all training sessions were carried out with loud background music.

### Statistical analysis

A two way repeated-measures ANOVA test followed by Bonferroni *post hoc* tests was used to examine the impact of training on performance variables while a one-way repeated-measures ANOVA was used to determine differences between pre- to post-test measures. All statistical tests were processed using the statistical package SPSS 18.0 (SPSS Inc, Chicago, IL). Statistical significance was set at p < .05. In addition, effect sizes (ES) were also calculated and interpreted using the following criterion: < 0.25 = trivial, 0.25–0.5 = small, > 0.5–1 = moderate and > 1 = large [21]. Test-retest reliability showed an intraclass correlation coefficient (ICC) for the 1RM bench press of 0.96 and a coefficient of variation (CV) of 3.5%, while ICC for power output measurements ranged from 0.84 to 0.92 with CV ranging from 7.9% to 10.2%. Standing throw velocity showed an ICC of 0.85 and CV of 3.3%, while ICC for the jumping throw velocity was 0.88 and CV 3.6%.

## Results

There were no significant between-groups differences at the pre-test measures in any variable of the study: bench press 1RM (p = .74), peak power at all RM percentages (p values ranging from .26 to .36), mean power at all RM percentages (p values ranging from .35 to .65), power at early time intervals at all RM percentages (p values ranging from .17 to .95), standing throw velocity (p = .89) and jumping throw velocity (p = .47).

The C group did not show any significant changes in maximal dynamic strength, power-related variables or throwing velocities over the 4 weeks of training.

### Maximal dynamic strength

Both the UL and the KL group showed significant increases in the 1RM bench press exercise (p < .01). The 1-RM increased from 76.2 ± 11.9 kg to 84.8 ± 13.7 kg (ES = 0.72; moderate) in the UL group, and from 74.6 ± 11.4 kg to 82 ± 13.8 kg (ES = 0.65; moderate) in the KL group.

### Peak power

Both UL and KL showed significant (p < .05) peak power improvements at 30% 1RM. (ES = 0.73, and 0.86; moderate; respectively) Nevertheless, no significant improvements were found at 50% 1RM nor at 70% 1RM (Table 3).

**Table 3:** Peak power output (W) in the three groups before and after the 4-week training intervention.

	30% 1RM		50% 1RM		70% 1RM	
	Pre	Post	Pre	Post	Pre	Post
**UL**	495 ± 88	560 ± 104*	564 ± 85	581 ± 111	504 ± 67	505 ± 86
**KL**	475 ± 87	550 ± 132*	537 ± 93	553 ± 109	470 ± 93	474 ± 112
**C**	523 ± 105	560 ± 147	549 ± 105	562 ± 95	501 ± 92	497 ± 107

* = significantly different from pre-test (p < .05).

### Mean power

The values of mean power output were significantly higher after the 4 weeks of training intervention in both UL (ES = 0.83; moderate) and KL (ES = 0.68; moderate) group at 30% 1RM. Moreover, only the UL group showed significant improvements in mean power values at 50% 1RM (ES = 0.47; small). No significant changes were found at 70% 1RM in either UL or KL groups (Table 4).

**Table 4:** Mean power output (W) in the three groups before and after the 4-week training intervention.

	30% 1RM		50% 1RM		70% 1RM	
	Pre	Post	Pre	Post	Pre	Post
**UL**	341 ± 60	391 ± 71*	367 ± 66	398 ± 81*	315 ± 47	333 ± 58
**KL**	323 ± 60	364 ± 59*	358 ± 62	376 ± 71	311 ± 51	324 ± 69
**C**	347 ± 85	362 ± 69	350 ± 69	376± 64	311 ± 43	310± 63

* = significantly different from pre-test (p < .05).

### Power in the early time intervals

**Fig 1:**
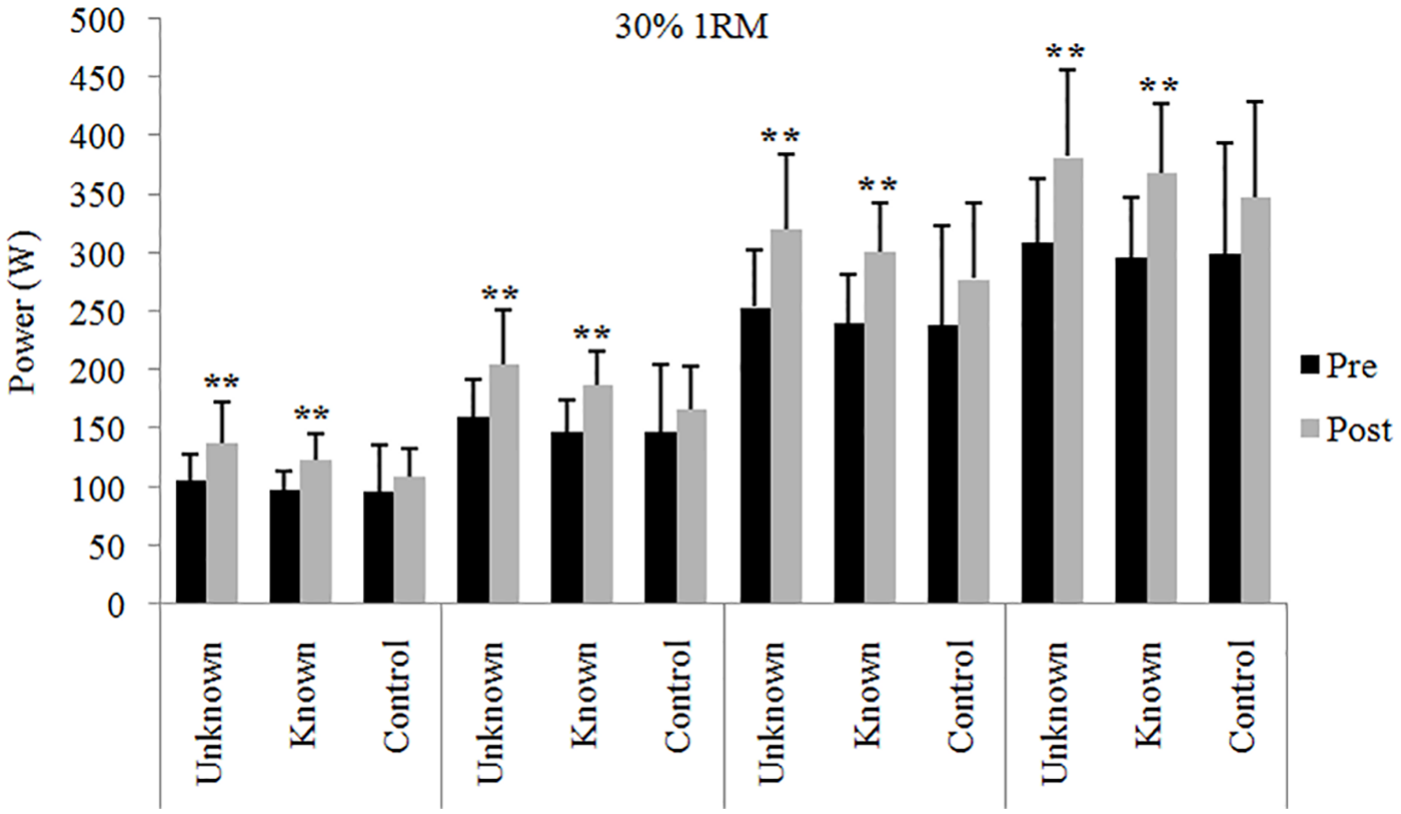
Power output at 30% 1RM. Power in the early time intervals with the 30% 1RM in the three groups before and after 4-week training intervention. ** = significantly different from pre-test (p < .01).

**Fig 2:**
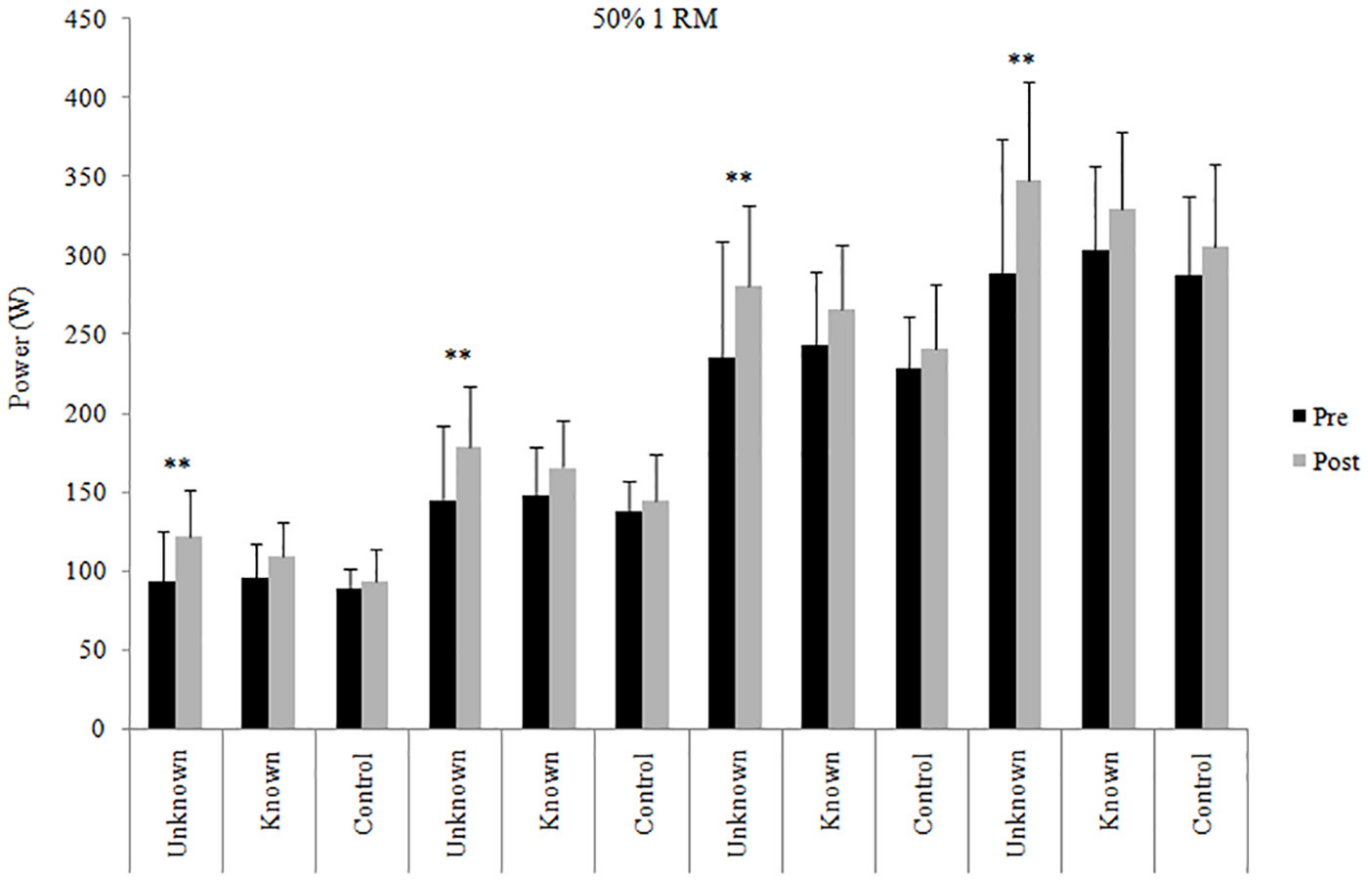
Power output at 50% 1RM. Power in the early time intervals at 50% 1RM in the three groups before and after 4-week training intervention. ** = significantly different from pre-test (p < .01).

**Fig 3:**
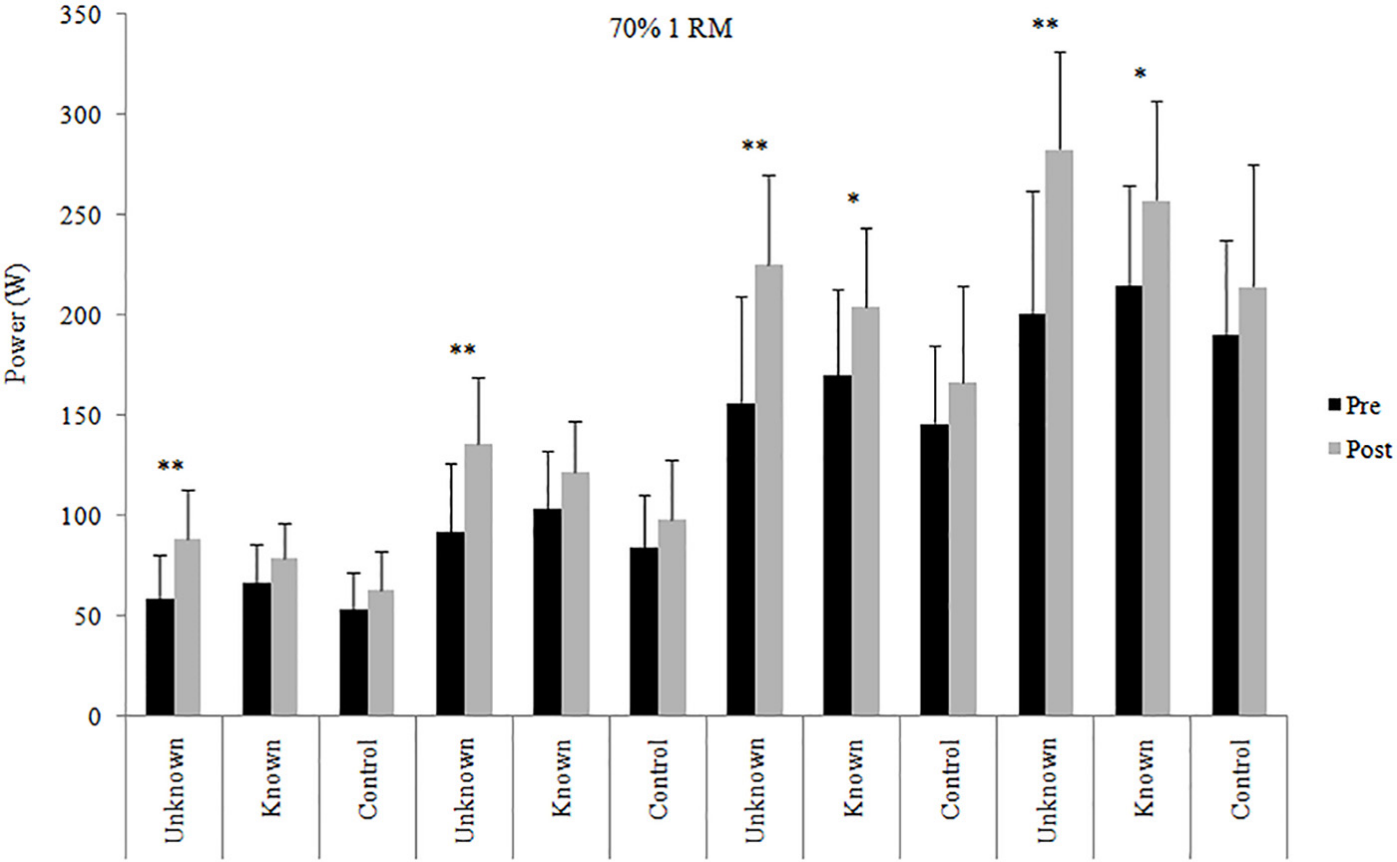
Power output at 70% 1RM. Power in the early time intervals at 70% 1RM in the three groups before and after 4-week training intervention. * = significantly different from pre-test (p < .05); ** = significantly different from pre-test (p < .01).

Data of power output in the early time intervals of the concentric phase before and after the 4 weeks of training intervention are shown in Figs 1, 2 and 3 (30%, 50% and 70% 1RM respectively).

Both the UL and KL groups showed significant (p < .01) increases in power output after the 4 weeks in the time intervals 30, 50, 100 and 150 ms at 30% 1RM (Fig 1). The increases in power developed in the early time intervals with the 30% 1RM were similar between groups. Thus, effect sizes were: 1.45, 1.41, 1.38 and 1.33 (at 30, 50, 100 and 150 ms), and 1.4, 1.35, 1.34 and 1.28 (at 30, 50, 100 and 150 ms) in UL and KL group respectively.

The power developed in the early time intervals pre- and post-training intervention at 50% 1RM is shown in Fig 2. Significant increases were found after training in the UL group at 30 ms (ES = 0.92; moderate), 50 ms (ES = 0.78; moderate), 100 ms (ES = 0.75; moderate) and 150 ms (ES = 0.91; moderate). The KL group did not show significant increases in power output at 50% 1RM at any time interval. Nevertheless, data of post-test showed a trend to significance in the KL group in all the early time intervals (p < .1) with ES ranging from 0.48 (small) to 0.61 (moderate).

In Fig 3 are shown data of power output in the early time intervals at 70% 1RM before and after the 4 weeks of training. After the training period, UL group showed significant higher power values in all early time intervals (p < .01), with large ES: 1.41 (0–30 ms), 1.39 (0–50 ms), 1.37 (0–100 ms) and 1.45 (0–150 ms). In addition, KL group showed increases in power output in the time intervals 0–100 ms (ES = 0.79; moderate) and 0–150 ms (ES = 0.84; moderate).

### Throwing velocity

After the 4-weeks training intervention, only the UL group showed significant (p < .05) increases in handball throwing velocity (Table 5). Specifically, standing throw velocity increased 4.7% (ES = 0.5; moderate) while the jumping throw increased 5.3% (ES = 0.51; moderate).

**Table 5:** Throwing velocities in the three groups before and after the 4-week training intervention.

	Standing throw (km·h^-1^)		Jumping throw (km·h^-1^)	
	Pre	Post	Pre	Post
**UL**	77.8 ± 7.6	81.6 ± 9.6*	82.1 ± 9	86.7 ± 9.5*
**KL**	76.5 ± 4.8	78.4 ± 6	78.4 ± 7.5	80.3 ± 7.3
**C**	80.8 ± 5.4	80.5 ± 3.7	84.3 ± 3.9	85.8 ± 3

* = significantly different from pretest (p < .05)

## Discussion

The purpose of the current study was to compare the strength, power, and handball throwing velocity changes following a 4-week strength training intervention based on unknown vs known loads. The main finding of this study was that after 4 weeks of training, the unknown load (UL) group improved the performance variables to a greater extent than the known load (KL) group. In this regard, only the UL group obtained greater power increases in the early time intervals in all load-ranges (30%, 50%, and 70% 1RM) and significant increases in both standing and jumping throw velocities.

It is known that explosive actions such as sprinting and jumping are largely related to the force produced in the early intervals (< 250 ms) [22,23]. In this particular case, handball throw movement has been shown to last approximately 180–240 ms [24], thus, increases in force production in the early time intervals of a movement are of significant practical use. This early force production can be increased following training interventions requiring the subject to contract ‘as fast and hard as possible’ [25,26], and with loads that are optimal for maximal power production [3,10]. However, whether these adaptations may be enhanced following unknown loads remained to be elucidated. The present study is the first to highlight the usefulness of this kind of stimulus (unknown loads) for improving power output in the early time intervals (i.e. 150 ms) of a movement. It has been reported that both a greater power output and muscle activation can be produced at early time intervals as an acute response of using unknown loads, when compared to known loads [13,27]. The mechanism by which the use of unknown loads supposes a greater stimulus to the central nervous system may be due to a possible overestimation of the weight, causing a larger force production than that required to move the real mass [28].Our results are in accordance with the acute studies, showing that the UL group improved to a larger extent when compared to the KL group (UL: + 14.3–33.7%; KL: 8.2–21.1%) in the power developed in the early time intervals (< 150 ms). The mechanisms underlying the greater improvements of the UL group could be explained by the subject’s response to lifting unknown loads, which has been previously reported as increased EMG response in pectoralis major and anterior deltoid muscles in the early time intervals before and after the movement onset [27], which is in agreement with the increases shown by several authors in muscle activation and movement velocities when using unknown loads compared with known loads [17,29]. The increases in power output in the early time intervals of the movement may be explained by both an increase in motor unit firing frequency [30], and increases in motor unit recruitment due to are duced recruitment threshold of the motor units [31].

Dynamic maximum strength is known to be improved by several strength training modalities and is of interest in many team sports. Our results showed that both the UL and the KL group (10.1% and 9% respectively) significantly improved their 1RM bench press. The increases in maximal force found in the present study are smaller than those shown by Hermassi et al. [6] after 8 weeks of training with heavy loads (16.8%), similar to those reported by Hermassi et al. [7] after 10 weeks of training with heavy loads (12.9%), and higher than those reached in such study with moderate loads (6.2%). Therefore, the current study has demonstrated that the use of a ballistic movement (bench press throw) with a combination of loads (30, 50, and 70% 1RM) may be an efficient way of increasing not only early force production, but also maximal dynamic strength, in a relatively short period of time (4 weeks). Due to the fact that pre-season in some sports (i.e. handball) allows a very limited amount of weeks to achieve strength/power adaptations, these increases after such short period of time are noteworthy.

In addition to the improvements in both power-related variables and maximal dynamic strength, it is of great interest to see whether these neuromuscular adaptations may translate to an enhancement of in-field performance (i.e., throwing velocity). Research showing if these neuromuscular adaptations transfer to the in-field performance are scarce, and mostly descriptive [22,23]. Our results show that this increase was significant only for the UL group in both the standing throw (4.7%) and jumping throw (5.3%), while the KL group showed a non-significant increase of 2.4%. It is important to highlight that similar improvements in throwing velocity after longer training interventions have been previously reported (after 8-week [7, 32], and 10-week [6]). It can be speculated that it is needed a certain amount of early force production improvement to see a transference to in-field performance, thus, being UK loads a more useful method to rapidly increase throwing performance.

## Conclusions

This study shows that 4 weeks of training intervention with unknown loads can enhance variables related to sports performance such as power output, and both standing and jumping throw velocity in time handball players. Given the importance of both power output and throwing velocity to team handball success, any training intervention leading to greater (or earlier) gains in this variables are of particular interest for coaches. The results of this study suggest that the use of unknown loads leads to greater improvements in such performance variables compared with known loads. Due to the limited weeks available in several sports’ preparatory period, these results are of practical relevance in terms of time-efficiency. Therefore, the use of unknown loads seems to offer a novel stimulus to the central nervous system leading to improvements in specific performance in sports where success is related to explosive force production, and especially when only a short period of training is possible.

## Supporting Information

S1 File Written informed consent. (DOCX)

S2 File Ethics statement. (DOCX)
