# Supplementary material for: Effects of 4-Week Training Intervention with Unknown Loads on Power Output Performance and Throwing Velocity in Junior Team Handball Players
Source: PLoS One. 2016 Jun 16;11(6):e0157648. doi: 10.1371/journal.pone.0157648 (PMC4911126; doi:10.1371/journal.pone.0157648)
Supplement: S2 File — (DOCX) [file pone.0157648.s002.docx]

SPECIFICATION FOR RESEARCH INVOLVING HUMAN NON INVASIVE PROCEDURES

| **Project title:** | *Influence of load knowledge in resistance training on rapid force production, muscle activation and strength gains* |
| --- | --- |

1 Introduction:

- The project evaluation criteria are based on Law 14/2007, of 3 July, on Biomedical Research.
- The items referred to in annex instruction and comments are those stated in this Law.

1. Funding:

None

1. Placement:

Sports Research Center (Universidad Miguel Hernández of Elche) and C.B. Elche sports facilities.

1. Staff of the project:

a) Main researcher:

Name and surname: Rafael Sabido Solana

Academic degree: PhD

Center: Sports Sciences

Department: Sports Research Center

Faculty: Universidad Miguel Hernández of Elche

Address: Avda. de la Universidad s/n 03202. Elche

Telephone number: 966.65.88.75 Fax: 966.65.86.97

Email: rsabido@umh.es

b) Other researchers:

Name and surname: Jose Luis Hernández Davó

Academic degree: MsC

Center: Sports Sciences

Department: Sports Research Center

Faculty: Universidad Miguel Hernández of Elche

Address: Avda. de la Universidad s/n 03202. Elche

Telephone number: 966.65.88.75 Fax: 966.65.86.97

Email: jlhdez43@gmail.com

Name and surname: Manuel Moya Ramón

Academic degree: PhD

Center: Sports Sciences

Department: Sports Research Center

Faculty: Universidad Miguel Hernández of Elche

Address: Avda. de la Universidad s/n 03202. Elche

Telephone number: 966.65.88.75 Fax: 966.65.86.97

Email: mmoya@umh.es

Name and surname: Javier Botella Ruiz

Academic degree: MsC

Center: Sports Sciences

Department: Sports Research Center

Faculty: Universidad Miguel Hernández of Elche

Address: Avda. de la Universidad s/n 03202. Elche

Telephone number: 966.65.88.75 Fax: 966.65.86.97

Email: javier.botella.ruiz@gmail.com

1. Aims:

The aim of this project is to check the influence of the use of unknown loads on (1) acute mechanical (power/RFD) and muscular (EMG) responses, and (2) the adaptations entailed after a training period using unknown loads.

Three studies will be carried out to develop this project.

a) Unknown loads affect force production capacity in early phases of bench press throws.

b) Load knowledge reduces rapid force production and muscle activation during maximal-effort concentric lifts.

c) Effects of 4-weeks training intervention with unknown loads on power output performance and throwing velocity in junior team handball players.

1. Methods:

Aiming at evaluating the influence of load knowledge on rapid force production, muscle activation, and strength gains, the following tests will be carried out in the studies:

*Maximal dynamic strength*

The 1-RM bench press was assessed using a previously established protocol (Earle, & Baechle, 2004), which requires that subjects progressively increase resistance across attempts until the 1RM is achieved. Rest period between trials was at least 5 minutes. Subjects began by lying horizontally with the buttocks, lower back, upper back and head firmly planted on the bench, with elbows fully extended and gripping the bar. Subjects lowered the bar until the chest was slightly touched, approximately 3 cm superior to the xiphoid process. The elbows were extended equally with the head and hips remaining in contact with the bench, and the feet in contact with the floor throughout the lift. No bouncing or arching of the back was allowed.

*Bench press throw power performance*

Three minutes after a warm-up consisting of two sets of 10 repetitions with the individuals 50% of 1RM, power performance was tested by performing one set of six repetitions of the bench press throw exercise using the individuals 30%, 50% and 70% 1RM. Rest period between sets was 5 minutes. An isoinertial dynamometer (T-Force Dynamic Measurement System, Ergotech, Murcia, Spain) was used for mechanical measurements. This system consists of a linear velocity transducer interfaced to a personal computer by means of a 14-bit resolution analog-to-digital data acquisition board, and custom software. Vertical instantaneous velocity was directly sampled by the device at a frequency of 1000 Hz. Instantaneous mechanical power output (P) was calculated as the product of vertical force and bar velocity (P = F · v). Peak power was taken as the maximum value of the power-time curve. The validity and reliability of this system have been previously established (González-Badillo, & Sánchez-Medina, 2010). All data were saved to disk for subsequent analysis. The variables analysed were: peak power, mean power, and power in the early time intervals (30, 50, 100 and 150 ms) of the concentric phase.

*Throwing velocity*

Handball throwing velocity was measured using a portable radar (Stalker sport 2, Applied Concepts Inc, USA) with an accuracy of 0.1 km·h^-1^. After 10 minutes of warm-up consisting of jogging, dynamic stretches and technical skills (passes and throws at submaximal velocities), each subject performed 3 maximal velocity throw attempts from both 7 m (standing throw) and 9 m (jumping throw). The standing throw has been described previously by Hermassi et al. (2010). In the jumping throw, players made a preparatory 3 step run before jumping vertically and releasing the ball while in the air, behind a line 9 m from the goal. The fastest throw of each throwing type was used for statistical analysis.

Assessment of EMG activity

EMG signals were recorded synchronously from anterior deltoid (1.5 cm distal and anterior to the acromion) and pectoralis major (4 cm medial to the axillary fold), both oriented in the predicted direction of the muscle fibers (Saetterbakken, & Fimland, 2013). In spite of the relevance of triceps brachii to the bench press movement, anterior deltoid has been shown to be more extensively activated (Anderson, & Behm, 2004; McCaw, & Friday, 1994) and was thus deemed most important for examination. The electrodes (11 mm contact diameter, 20 mm center-to-center distance) were placed on the side of the dominant (strongest) arm (Saetterbakken, & Fimland, 2013). The skin was prepared (shaved, abraded and cleaned with alcohol) before the placement of pre-gelled disposable bipolar Ag/AgCl surface electrodes (Arbo Infant Electrodes, Tyco Healthcare, Germany). A commercial EMG recording system (Muscle Tester ME6000, Mega electronics Ltd., Kuopio, Finland) and microcomputer with an 8-channel A/D conversion (14-bit resolution) and common mode rejection exceeding 110 dB was used to record the EMG signals at an analog-to-digital conversion rate of 1000 Hz. The signals were high- (500 Hz; Butterworth 2^nd^ order) and low-pass (8 Hz; Butterworth 4^th^ order) filtered. The raw EMG signals were smoothed with a symmetric root-mean-square (RMS) filter with a 50-ms time constant and transferred via an optical cable to a compatible computer where it was monitored using a dedicated software (Megawin 2.5, Mega Electronics Ltd., Kuopio, Finland).

An electrogoniometer was placed on the arm-forearm to determine movement onset, defined as the point at which the elbow joint exceeded 3 times the standard deviation of the baseline variability; this point was also checked visually in all trials. Direct measurement at the elbow was deemed more accurate than the measurement of bar movement onset because the latter is influence by the delay in inter-joint mechanical coupling in the arm (i.e. delay between proximal joint rotation and displacement of the bar). Before testing, each subject performed two maximal voluntary isometric contractions (MVIC) separated by 2 min with an elbow joint angle of 90º. The greater MVIC trial was used to normalize the muscle activation during the bench press throws. EMG data were analyzed over the following time intervals: pre100 - pre50 ms, pre50 - 0 ms (where 0 corresponds to the movement onset), 0 - 50 ms, 50 - 100 ms and 100 - 150 ms. Additionally, the normalized change in RMS EMG between the different time intervals was used as a measure of the rate of EMG rise (RER) (Aagaard et al., 2002; Barry, Warman, & Carson, 2005; Blazevich et al., 2008). All data were stored for offline analysis with custom-built software (LabView, National Instruments, Austin, Texas). As EMG data were collected in both conditions within a single session and under identical (and non-fatiguing) measurement conditions, we considered the amplitude of the EMG signal to provide an estimate of the level of muscle activation by the central nervous system.

For the analysis of latencies between the EMG onsets and the beginning of the movement, both pectoralis major and anterior deltoid EMG baseline activities were calculated over a 300 ms window. The EMG onset was defined as the moment at which a value of the respective signal rose above 3 times the standard deviation from the baseline mean. All onsets were also visually verified to ensure accuracy.

6.2 Project duration:

2 years

6.3 Fast:

| x | No | | | |  |  |
| --- | --- | --- | --- | --- | --- | --- |
|  | Yes: | | | |  |  |
| Study phase | | Food | Water | Start | Final | Duration (h) |
|  | |  |  |  |  |  |

6.4 Product administration:

| x | No | | | | |
| --- | --- | --- | --- | --- | --- |
|  | Yes: | | | | |
| Name | | Via | Volume ml/kg | Dose mg/kg | Concentration mg/ml |
| Product | |  |  |  |  |

6.5 Blood samples:

| x | No | | |
| --- | --- | --- | --- |
|  | Yes: | | |
| Via | | Volume (ml) | Frequency of extraction |
|  | |  |  |

1. Phases in which pain is expected and methods to control and limit it:

7.1 Describe stages of the proceedings when is expected that the individual can experience suffering, pain or distress and that measures be taken to control and limit.

No pain or distress is expected in the studies

7.2 Indicate that maximum degree of severity that can be achieved:

| 0 | 1 | 2 | 3 | 4 |
| --- | --- | --- | --- | --- |
| x |  |  |  |  |

7.3 Supervision protocol:

| - What is going to be controlled: the subjects will be asked whether they feel good or if they feel any pain during activities. Finally, they can choose to stop the activity when any sensation of pain, dizziness or distress appears. |
| --- |
| - When: Since the beginning of the recording sessions. |
| - For how long: Throughout the process involving the performance of physical tests. |
| - Frequency of controls: The controls will be continuous during the performance of physical tests. |
| - Researcher in charge: All researchers of the project |

7.4 Anesthetic:

¿Is going to be used?

| x |  |  |  |  |  |
| --- | --- | --- | --- | --- | --- |
|  |  |  |  |  |  |
| Phase | | Product name | Via | Dose mg/kg | Concentration |
|  | |  |  |  |  |

7.5 Analgesic products:

¿Are going to be used?

| x | No | | | | | |  |
| --- | --- | --- | --- | --- | --- | --- | --- |
|  | Yes | | | | | |  |
| Phase | | Product name | Vía | Dose mg/kg | Concentration mg/ml | Frequency | Duration |
|  | |  |  |  |  |  |  |

1. Procedures intended to safeguard the confidentiality of the data:

The data obtained in this study will be specifically addressed by the research group, which shall at all times by the anonymity and integrity of the participants, obeying the fulfillment of the Law 15/1999 on Protection of Personal Data. To this end, each participant will be assigned a registration number that will serve to the development of databases and the processing and analysis of data. No person, except members of the research group, will know the results obtained by the participants in the different tests and records.

1. Compliance with the law:

Therefore, as a researcher / a responsible for this project, says that:

- Has studied and evaluated the existence of alternative methods, and has not identified the existence of an alternative method or protocol for the outcome of the proposed study.

- All individuals undergoing invasive procedure are previously insured of damages that might result from it, as stated in Article 18 of Law 14/2007 of 3 July, on Biomedical Research.

- The above is in accordance with the law and, in general, with all applicable legislation.
